# Joint efficacy of the three biomarkers *SNCA*, *GYPB* and *HBG1* for atrial fibrillation and stroke: Analysis via the support vector machine neural network

**DOI:** 10.1111/jcmm.17224

**Published:** 2022-02-09

**Authors:** Xiang Wang, Xuyang Meng, Lingbing Meng, Ying Guo, Yi Li, Chenguang Yang, Zuowei Pei, Jiahan Li, Fang Wang

**Affiliations:** ^1^ Department of Cardiology Beijing Hospital National Center of Gerontology Institute of Geriatric Medicine Chinese Academy of Medical Sciences Beijing China; ^2^ Graduate School of Peking Union Medical College Chinese Academy of Medical Sciences Beijing China; ^3^ The First Mobile Corps of People's Armed Police Beijing China

**Keywords:** atrial fibrillation, *GYPB*, *HBG1*, molecular function, *SNCA*, stroke

## Abstract

Atrial fibrillation (AF) is the most common type of persistent arrhythmia. Although its incidence has been increasing, the pathogenesis of AF in stroke remains unclear. In this study, a total of 30 participants were recruited, including 10 controls, 10 patients with AF and 10 patients with AF and stroke (AF + STROKE). Differentially expressed genes (DEGs) were identified, and functional annotation of DEGs, comparative toxicogenomic database analysis associated with cardiovascular diseases, and predictions of miRNAs of hub genes were performed. Using RT‐qPCR, biological process and support vector machine neural networks, numerous DEGs were found to be related to AF. *HBG1*, *SNCA* and *GYPB* were found to be upregulated in the AF group. Higher expression of hub genes in AF and AF + STROKE groups was detected via RT‐PCR. Upon training the biological process neural network of *SNCA* and *GYPB* for *HBG1*, only small differences were detected. Based on the support vector machine, the predicted value of *SNCA* and *GYPB* for *HBG1* was 0.9893. Expression of the hub genes of *HBG1*, *SNCA* and *GYPB* might therefore be significantly correlated to AF. These genes are involved in the incidence of AF complicated by stroke, and may serve as targets for early diagnosis and treatment.

## 1 INTRODUCTION

Atrial fibrillation (AF) is the most common cardiac arrhythmia,^1^ affecting 33 million people worldwide annually.^2^ Given the association between AF, obesity, hypertension and other rapidly growing health problems, its prevalence is projected to double over the next 30 years.^3^ AF is associated with a threefold to fivefold increased risk of stroke,^4^ and AF‐related strokes are typically severe, causing significant long‐term physical disability and cognitive dysfunction, high mortality and healthcare costs, compared to other stroke subtypes.^5^ However, the molecular mechanisms underlying stroke caused by AF are unclear.^6^ The risk factors for AF include old age, primary hypertension, heart failure, myocardial infarction, structural cardiac diseases, obesity, diabetes mellitus, obstructive sleep apnoea and exposure to toxicants,^7^, ^8^, ^9^, ^10^ none of which can accurately predict the occurrence and development of AF. Therefore, identifying the predictors of AF and AF‐related stroke is a focus of current efforts. Studying the genetic factors responsible for stroke caused by AF is vital for understanding its pathogenesis, and providing a theoretical foundation for precise treatment.^11^, ^12^, ^13^

In this study, three datasets, GSE75092, GSE64904 and GSE58294, were downloaded from the Gene Expression Omnibus (GEO), followed by screening and enrichment of differentially expressed genes (DEGs) and identification of hub genes. Finally, we conducted a bioinformatic analysis of DEGs and predicted microRNAs (miRNAs) relevant to patients with AF prone to stroke.

## 2 MATERIALS AND METHODS

### 2.1 Datasets

Gene Expression Omnibus is a gene expression database created and maintained by the National Center for Biotechnology Information. It contains high‐throughput gene expression data submitted by research institutions worldwide. We obtained the transcriptome expression profiles GSE64904, GSE58294 and GSE75092 from GEO. The probes were transformed into homologous gene symbols using the annotation information of the platform. The GSE64904 dataset contained 3 AF samples and 3 control samples; GSE58294 contained 69 AF with stroke samples and 23 control samples; and GSE75092 contained 6 AF samples and 3 control samples.

### 2.2 Repeatability test for the datasets

Principal component analysis (PCA) is a statistical method; through orthogonal transformation, a group of variables that may be correlated is transformed into a group of linearly unrelated variables, which are called principal components. PCA was used in this study to test the repeatability of the GSE64904, GSE58294 and GSE75092 datasets. In addition, Pearson's correlation test was performed to verify intra‐group data repeatability in each group. The R programming language is an operating environment for statistical analysis and graph plotting, and was used to visualize the correlation between every sample from the same dataset using heat maps.

### 2.3 Screening of differently expressed genes (DEGs)

The Linear Models for Microarray Data (‘Limma’) R package not only contains RAW data input and pre‐processing (normalization) functions for cDNA chip data, but also the ‘linear’ algorithm of differentiated gene analysis, especially for a ‘multifactor designed experiment’. The Limma package is very scalable and can analyse differential genes from either one‐channel or two‐channel data. It offers many options for data loading, data pre‐processing (background correction, intra‐group normalization and inter‐group normalization) and differential gene analysis.

The DEGs were screened using Limma; the cut‐off criteria for GSE64904 were *p* < 0.05, and a fold change (FC) ≥2 or ≤−2. The cut‐off criteria for GSE58294 were *p* < 0.05, and FC ≥0.5 or ≤−0.5. The cut‐off criteria in GSE75092 were *p* < 0.05, and FC ≥3 or ≤−3. Volcano maps were drawn using the volcano plotting tool (https://shengxin.ren) to identify DEGs.

### 2.4 Construction and analysis of protein‐protein interaction (PPI) network

The Search Tool for the Retrieval of Interacting Genes (STRING) database (https://string‐db.org/) is an online search database for known protein interactions and was used for the construction of the PPI network. Currently updated to version 10.5, it stores information on 2031 species, 9,643,763 proteins and 1,380,838,440 interactions.

### 2.5 Identification of the hub genes

Cytoscape software is used for biological network analysis and two‐dimensional (2D) visualization. In our study, the PPI network constructed using the STRING database was analysed using Cytoscape. The hub genes were excavated when the degrees were set to ≥10. Three other algorithms (EPC, MCC and MNC) were used to identify the hub genes. Next, molecular complex detection (MCODE) (version 1.5.1, a plug‐in of Cytoscape) was used to identify the most important module of the network map. The criteria of MCODE analysis were as follows: degree cut‐off =2, MCODE scores >5, Max depth =100, node score cut‐off =0.2 and k‐score =2. Venn diagrams were delineated using the online VENN tool (http://bioinformatics.psb.ugent.be/webtools/Venn/), which could visualize common DEGs.

### 2.6 Sequence comparison of long intergenic non‐coding RNAs (LincRNA) and genes

BLAST is the primary tool used in NCBI to compare a protein or DNA sequence with other sequences in various databases. The LincRNAs in GSE75092 that were differentially expressed between atrial fibrillation and control were identified, and the LincRNA and gene sequences were compared using BLAST.

### 2.7 Functional annotation of DEGs

Gene Ontology (GO) analysis annotates the functions of genes using terms from a dynamic, controlled vocabulary based on three aspects of biology: biological processes (BP), cellular components (CC) and molecular functions (MF). Kyoto Encyclopedia of Genes and Genomes (KEGG) (http://www.genome.jp) contains information about specific pathways and links genomic information with higher‐order functional information. Gene set enrichment analysis (GSEA) (http://www.broadinstitute.org/gsea/downloads.jsp) is a computational method that conducts GO and KEGG analyses for a given gene list, and is used to analyse genome‐wide expression profiling data on a chip. Based on the existing knowledge of gene localization, function and biological significance, a molecular label database containing several gene sets was constructed. By analysing gene expression data, we determined whether the expression status was significantly enriched in a certain function.

Metascape integrates many authoritative data resources, such as GO, KEGG, UniProt and DrugBank, to perform pathway enrichment, biological process annotation, gene related protein network analysis and drug analysis.

The Database for Annotation, Visualization and Integrated Discovery (DAVID) v.6.8 comprises a full Knowledgebase update to the sixth version of our original web‐based programs. DAVID now provides a comprehensive set of functional annotation tools for investigators to understand the biological meaning behind a large list of genes.

Gene set enrichment analysis, Metascape, BINGO and DAVID are online analysis tools that provide a comprehensive gene list annotation and analysis resource. In our study, GO and KEGG analyses of DEGs were performed using GSEA, Metascape and DAVID (*p* < 0.05).

### 2.8 Identification of hub genes associated with cardiovascular diseases

CTD is a web‐based database that can identify relationships between genes, proteins and diseases. In our study, the relationship between gene products and cardiovascular diseases was analysed using this database.

### 2.9 Prediction of miRNAs of hub genes

TargetScan is an analysis tool that can perform predictive analyses, and determine possible mechanisms for the co‐regulation of the expression of hundreds of genes expressed in different cell types. In our study, miRNAs that regulate hub genes were screened using TargetScan.

### 2.10 RT‐qPCR

Total RNA was extracted from the blood samples using TRIzol^®^ (Beijing Biolab Technology Co., Ltd.) and reverse transcribed into cDNA using the Servicebio^®^RT First Strand cDNA Synthesis kit (cat. no. G3330, Wuhan Servicebio Biotechnology Co., Ltd.) for 60 min at 42°C. The reaction was terminated by heating at 70°C for 5 min. RT‐qPCR was performed in a Light Cycler^®^ 4800 System (Roche Diagnostics) with a specific set of primers for the amplification of the hub genes. The primers used are shown in Table S1. The thermocycling conditions were as follows: 95°C for 15 s followed by 60°C for 60 s (a total of 30 cycles). The relative quantification units (relative quantification = 2^−ΔΔCt^, where Ct represents quantification cycle values) of each sample, were calculated and presented as fold change of gene expression relative to the control group. *GAPDH* was used as an endogenous control.

### 2.11 BP neural network

The BP neural network is a type of multilayer feed‐forward neural network, which is characterized by signal forward transmission and error back propagation. In the forward transmission, the input signal is processed layer by layer from the input layer to the output layer. The state of the neurons in each layer only affects the state of the neurons in the next layer. If the output layer cannot obtain the expected output, it uses back propagation to adjust the weights and width of the neural network according to the prediction error, so that the predicted output constantly approaches the expected output.

The basic algorithm of the BP neural network is as follows:

(1) All weights are given randomly {*w_ji_*}{*v_ih_*}, including the threshold, usually with in {−1,1}.

(2) According to the K sample {*s*(*k*)}, calculate

(1) $$net_{i}^{A}\left(k\right)=\sum_{h=1}^{n+1}v_{\mathrm{ih}}s_{h}\left(k\right),i=1\ldots p$$

(2) $$a_{i}\left(k\right)=\sigma \left(net_{i}^{A}\left(k\right)\right),i=1\ldots p$$

Among them *s_n_*_+1_ (*k*) = 1,*v_in_*_+1_=*θ_i_*

(3) Calculation

(3) $$y_{j}\left(k\right)=\sum_{j=1}^{p+1}w_{\mathrm{ji}}\left(k\right)a_{i}\left(k\right),j=1\ldots q$$

Among them *w_jp_*_+1_ = *r_j_*

(4) Calculation

(4) $$e_{\mathrm{yj}}\left(k\right)=d_{j}\left(k\right)‐y_{j}\left(k\right),j=1\ldots q$$

(5) $$e_{\mathrm{ai}}\left(k\right)=a_{i}\left(k\right)\left(1‐a_{i}\left(k\right)\right)\sum_{j=1}^{q}w_{\mathrm{ji}}e_{\mathrm{yj}}\left(k\right),j=1\ldots q$$

(5) Training {*w_ji_*}{*v_ih_*}

(6) $$\begin{array}{c} w_{\mathrm{ji}}\left(k+1\right)=w_{\mathrm{ji}}\left(k\right)‐\eta a_{i}\left(k\right)e_{\mathrm{yj}}\left(k\right),i=1\ldots p+1,j=1\ldots q\\ v_{\mathrm{ih}}\left(k+1\right)=v_{\mathrm{ih}}\left(k\right)‐\eta e_{\mathrm{ai}}\left(k\right)s_{h}\left(k\right),i=1\ldots p+1,h=1\ldots n+1 \end{array}$$

(6) Go back to (2) and reiterative training.

### 2.12 SVM neural network

Support vector machine (SVM), first proposed by Vapnik, can be used for pattern classification and nonlinear regression, such as for multilayer perceptron networks and radial basis function networks. The main idea of the SVM is to establish a classification hyperplane as a decision surface to maximize the isolation edge between positive and negative cases. The theoretical basis of SVM is statistical learning theory; more precisely, SVM is the approximate realization of structural risk minimization.

This principle is based on the fact that the error rate of the learning machine on the test data (generalization error rate) is bounded by the sum of the training error rate and a term that depends on the Vapnik–Chervonenkis (VC) dimension. In the separable mode case, the SVM treats the first term as zero and minimizes the second term. Therefore, although it does not take advantage of the intra‐domain problems, a property unique to SVM is that it shows good generalization performance on pattern classification problems.

SVMs have the following advantages:

1. Generality: can be applied to a wide range of situations;
2. Robustness: no fine tuning is required;
3. Effectiveness: one of the best methods for solving practical problems;
4. Simple calculation: the method requires very simple optimization technology;
5. Theoretical perfection: based on the VC extensibility theory framework.

The concept of the inner product kernel between ‘support vector’ x (i) and the input space extracted vector x is the key to constructing the learning algorithm for SVMs. The supporting disc machine is composed of a small subset extracted from the training data using the algorithm.

The architecture that supports directional machines is shown in Figure 2.

### 2.13 Statistical methods

Strong correlations among genes were determined using the BP neural network and SVM. All statistical analyses were conducted using SPSS software (version 24.0; IBM Corp., Armonk, NY, USA) and MATLAB (MathWorks Inc., USA, R2017a). Statistical significance was set at *p* < 0.05.

## 3 RESULTS

### 3.1 **Validation of the** GSE64904 **dataset**

**FIGURE 1:**
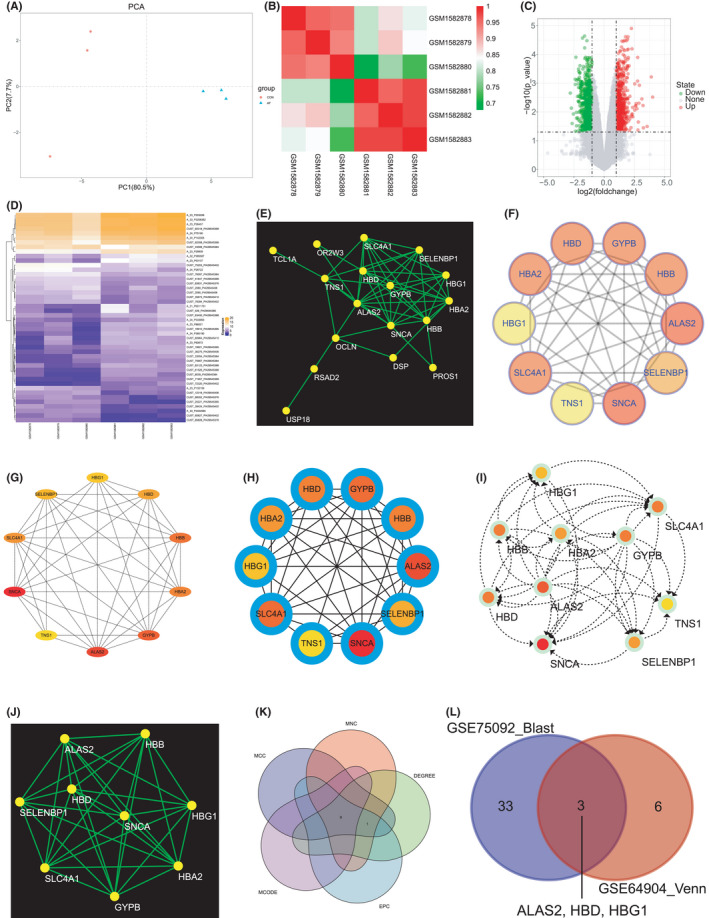
Validation of the GSE64904 dataset, and identification of the DEGs and the hub genes between AF and control

Principal component analysis analysis indicated that the intra‐group data repeatability was acceptable in GSE64904. The distances between samples in the control group were short, and the distances between samples in the AF group were also short in the PC1 dimension (Figure 1A). Furthermore, Pearson's correlation test indicated strong correlations among the samples in the AF and strong correlations among the samples in the control group in GSE64904 (Figure 1B).

### 3.2 Identification of the DEGs and the hub genes

The volcano map shown in Figure 1C indicates numerous DEGs between the AF and control groups; the green plots represent the downregulated DEGs, and the red plots represent the upregulated DEGs. The heatmap (Figure 1D) indicates significant differences in the expression levels of DEGs between the AF and control groups. The PPI network of DEGs was constructed using the STRING online database and analysed using Cytoscape software (Figure 1E). Five different algorithms, namely Degreen (Figure 1F), EPC (Figure 1G), MCC (Figure 1H), MNC (Figure 1I) and MCODE (Figure 1J), were employed to identify the hub genes. Nine hub genes (*HBG1*, *HBD*, *HBA2*, *ALAS2*, *SELENBP*, *SNCA*, *SLC4A1*, *HBB* and *GYPB*) were identified using a Venn diagram (Figure 1K). Furthermore, three common hub genes (*ALAS2*, *HBD* and *HBG1*) were identified between GSE75092 and GSE64904 (Figure 1L).

### 3.3 Difference the expression of hub genes in AF and control groups

**FIGURE 2:**
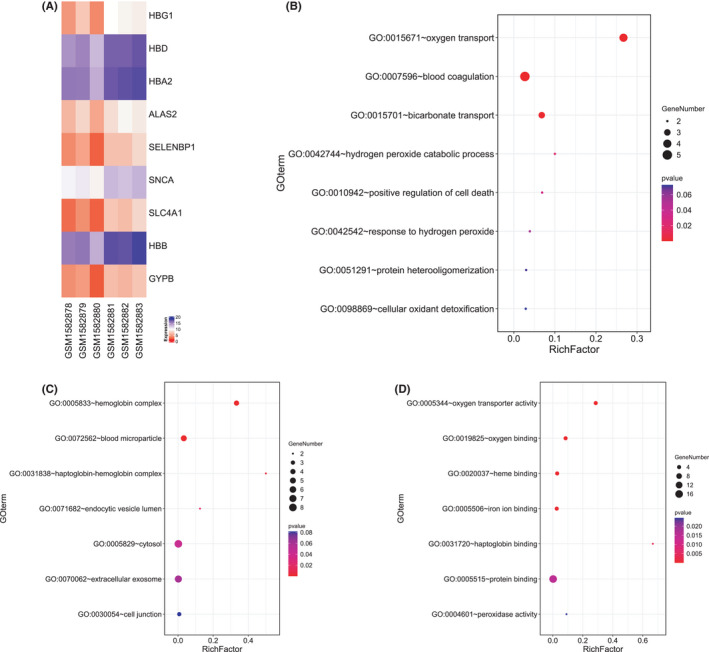
Expression difference of hub genes between AF and control, and enrichment analysis by DAVID

The heatmap showed that the expression of hub genes was upregulated in the AF group compared with that in the control group (Figure 2A).

### 3.4 Functional and pathway enrichment analysis of DEGs

The enrichment results of the GO and KEGG analyses of DEGs, obtained using DAVID, were mainly enriched in ‘oxygen transport’, ‘blood coagulation’, ‘positive regulation of cell death’, ‘cellular oxidant detoxification’, ‘hemoglobin’, ‘blood microparticle’, ‘haptoglobin‐hemoglobin complex’, ‘endocytic vesicle lumen’, ‘cytosol’, ‘extracellular exosome’, ‘cell junction’, ‘oxygen transporter activity’, ‘oxygen binding’, ‘heme binding’, ‘iron ion binding’ and ‘protein binding’ (Figure 2B–D).

### 3.5 GSEA analysis for the KEGG enrichment

**FIGURE 3:**
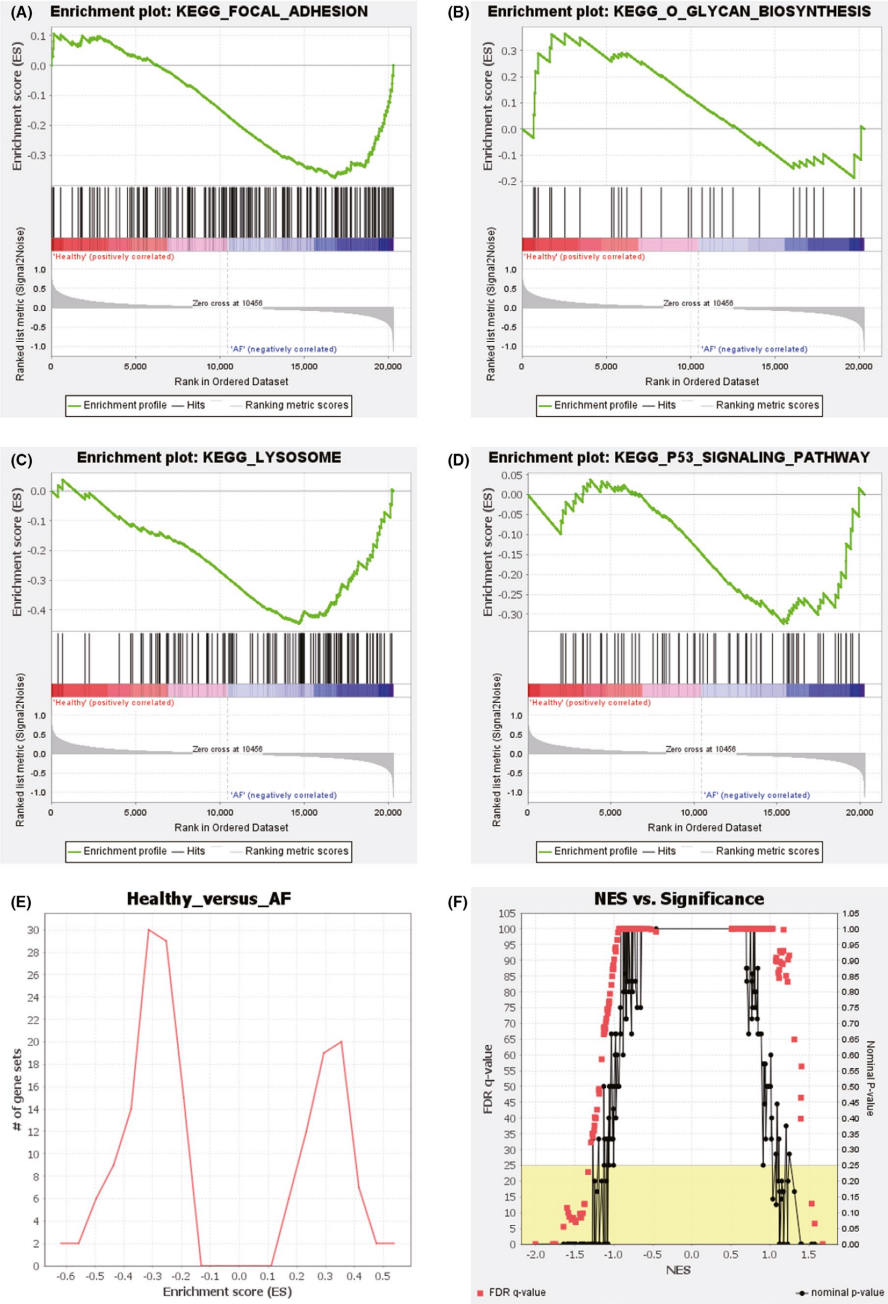
GSEA analysis for KEGG enrichment. (A) Focal adhesion. (B) Glycan biosynthesis. (C) Lysosome. (D) P53 Signalling pathway. (E) Enrichment score. (F) Normal enrichment score vs. significance

The KEGG analysis of DEGs obtained using GSEA showed that they were mainly enriched in ‘focal adhesion’, ‘O glycan biosynthesis’, ‘lysosome’ and ‘P53 signaling pathway’ (Figure 3A–D). The comparison between AF and control was fine based on the enrichment score and significance (Figure 3E–F).

### 3.6 Hub gene functions

**FIGURE 4:**
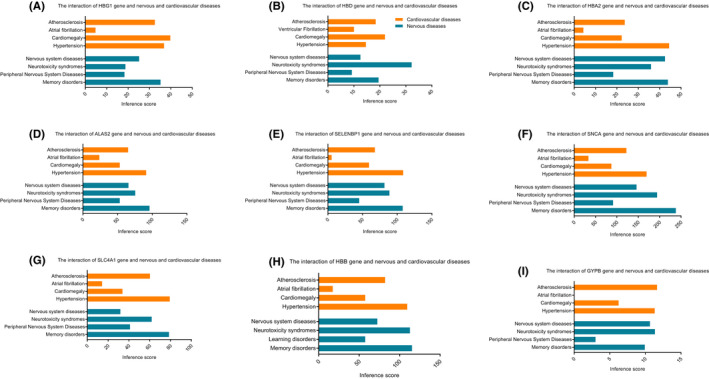
Identification of hub genes via the CTD database

Analysis of the CTD database indicated that the hub genes targeted cardiovascular diseases and neural system diseases, as shown in Figure 4.

### 3.7 **Elevated expression of hub genes in the AF and AF** **+ STROKE Groups**

**FIGURE 5:**
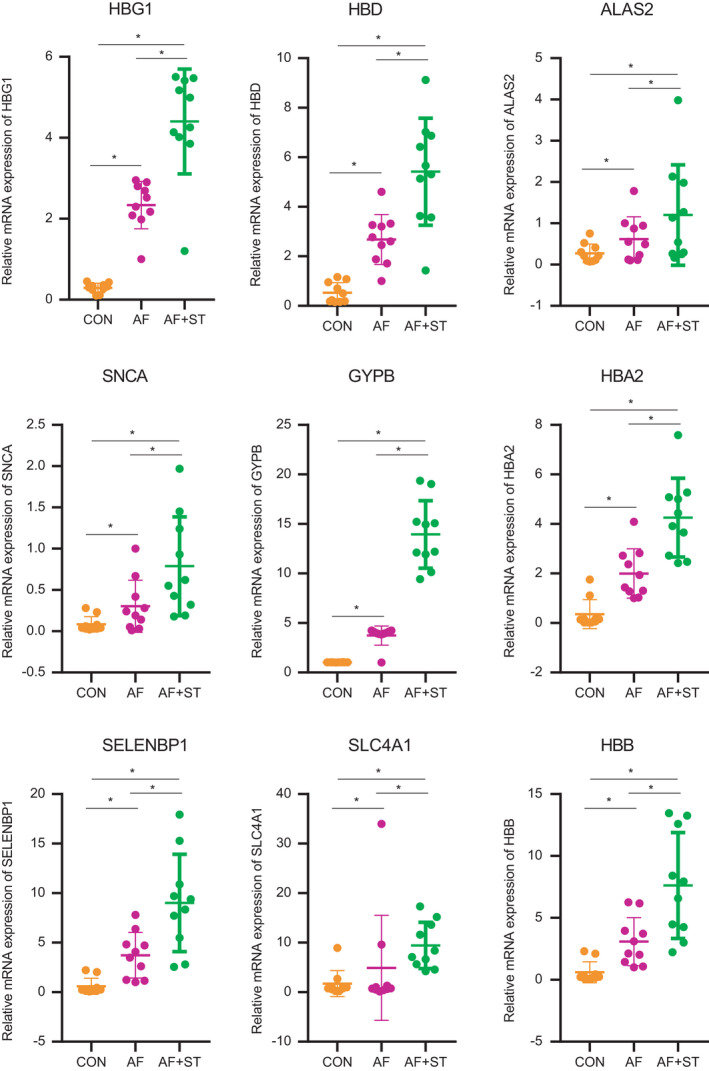
Upregulation of hub genes in the AF and AF + STROKE groups

The expression of hub genes in the AF group was higher than that in the control group (*p* < 0.05). Furthermore, the expression of hub genes in the AF + STROKE group was upregulated compared to that in the AF group (*p* < 0.05). (Figure 5).

### 3.8 Neural network prediction model and high‐risk warning range of SNCA, GYPB, and HBG1

**FIGURE 6:**
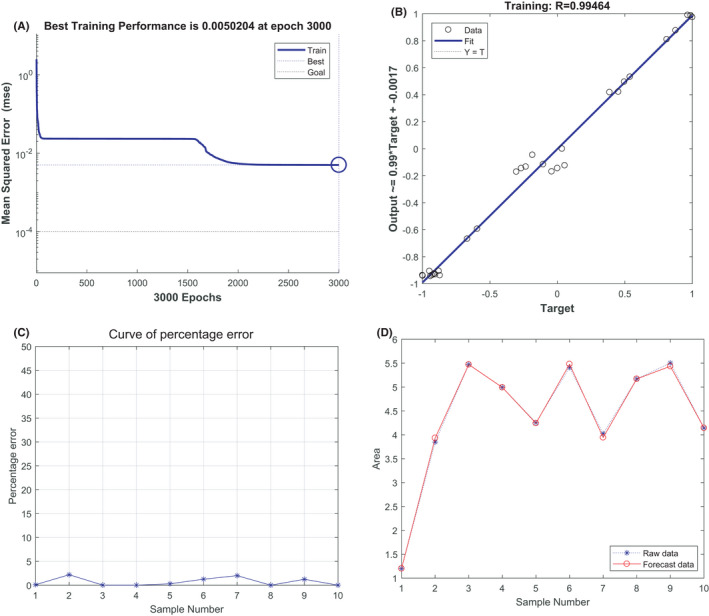
Neural network prediction model and high‐risk warning range of *SNCA*, *GYPB*, and *HBG1*

The BP neural network of *SNCA* and *GYPB* was trained for *HBG1*; the best training performance was 0.018092 at epoch 2999 (Figure 6A), and the relativity was 0.96057 (Figure 6B). Verification of the forecast data against the raw values indicated only minor differences (Figure 6C, D). Based on the above results, we speculated that the expression of SNCA and GYPB might be predictive indexes of HBG1.

### 3.9 Predictive value of SNCA and GYPB for HBG1 via SVM

**FIGURE 7:**
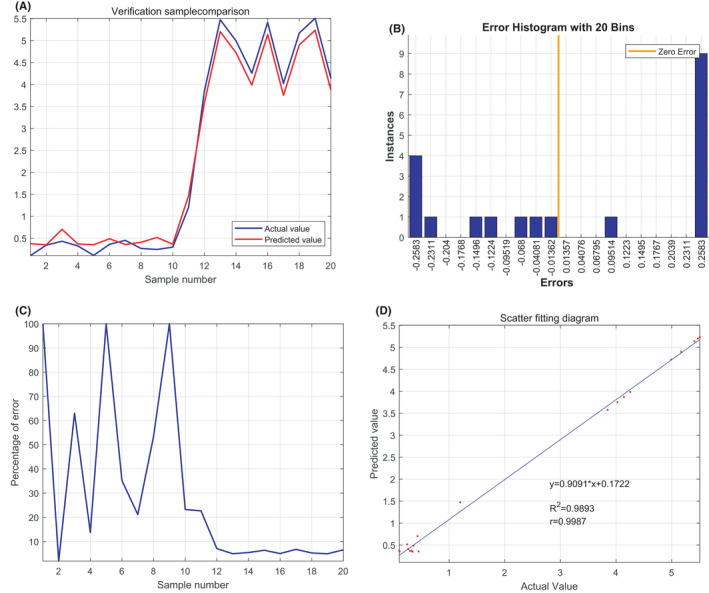
Predictive value of *SNCA* and *GYPB* for *HBG1* via support vector machine (SVM)

Support vector machine predicted values of SNCA and GYPB for HBG1 were 0.9893 (y = 0.9091*x + 0.1722), and the mean error was small (Figure 7).

## 4 DISCUSSION

Atrial fibrillation is a common and highly preventable cause of stroke. AF‐associated strokes are severe, with a high risk of recurrence, and may be the first clinical manifestation of AF.^14^ Furthermore, even in the absence of clinically manifest AF, subclinical AF is suspected to be the underlying cause for a large proportion of the 20%–25% of ischaemic strokes that are deemed ‘cryptogenic’.^15^ This is particularly concerning, given that patients who experience AF‐associated stroke have worse outcomes than those who experience stroke not related to AF.^16^, ^17^ Despite rapid advances in AF research, effective approaches for diagnosing and treating AF and AF‐associated strokes are still lacking at the molecular level. Bioinformatic analysis has been widely used to discover genetic changes responsible for disease occurrence and development, and is a reliable means of identifying targets for disease diagnosis or treatment.^18^

Our bioinformatic analysis showed that in the GSE64904 and GSE75092 datasets, *ALAS2*, *HBG1* and *HBD* genes were significantly and highly expressed in the AF group compared to the non‐AF group. In the GSE64904 and GSE58294 datasets, *SNCA* and *GYPB* genes were significantly and highly expressed in the AF and AF + STROKE group, compared to the non‐AF group. DEGs were significantly enriched in haem binding, oxygen transporter activity and blood coagulation. Clinical sample validation showed that the expression of hub genes in the AF group was higher than that in the control group. Furthermore, the expression of hub genes in the AF + STROKE group was upregulated compared to that in the AF group. Upon training the BP neural network of *SNCA* and *GYPB* for *HBG1*, the best training performance was 0.018092 at epoch 2999, and the relativity was 0.96057. The BP neural network was verified by SVM.

*ALAS2*, which catalyses the initial, rate‐limiting step during haem biosynthesis, is an erythroid‐specific mitochondrial gene. The expression of *ALAS2* during erythroid differentiation is strongly activated to meet the demand for haemoglobin. Haem is an essential iron‐containing metabolite for aerobic organisms and serves as a prosthetic group for haemoproteins involved in numerous cardiovascular processes, including oxygen transport, oxygen storage, oxygen metabolism, anti‐oxidation, electron transport, signalling and enzyme catalysis.^19^, ^20^ Previous studies have shown that *ALAS2* mutations prevent RBC differentiation due to haem deficiency during the proerythrocyte phase, thereby causing severe anaemia.^21^ Studies have shown that *ALAS2* is induced in the failing human heart, particularly in patients with ischaemic cardiomyopathy, and mechanistic studies show that *ALAS2* overexpression in cultured cardiomyoblasts results in mitochondrial oxidative stress and cell death through increased haem accumulation.^22^, ^23^

In this study, *HBG1* and *HBD* were identified as hub genes. *HBD*, encoding the unique d‐globin chain of HbA2, arose via duplication of the *HBB* gene after the marsupial/eutherian split, and is therefore unique to placental mammals.^24^ *HBG1* and *HBD* are important genetic components of haemoglobin β‐globin.^25^ Haemoglobin (Hb), a protein highly expressed in red blood cells, is a major oxygen‐transporting molecule that plays a key role in cellular aerobic metabolism. Human Hb is a tetramer composed of two α‐like and two β‐like globin chains that are covalently linked to haem, the oxygen‐binding group. *HBG1* induces erythroid foetal haemoglobin expression and reduces morbidity and mortality from haemoglobin diseases.^26^ *HBD* has been found to be closely associated with inflammation,^24^ and upregulation of *HBD* has been observed during infection and inflammation.^27^ In agreement with previous studies, the GO annotations related to *HBD* are oxygen transport, iron ion binding, blood coagulation and combination with oxygen.^28^ Sickle cell disease (SCD) and β‐thalassemia are the two most prevalent β‐haemoglobinopathies worldwide.^29^ A unifying feature of this heterogeneous group of diseases is reduced functional haemoglobin due to altered protein structure in SCD, or insufficient β‐haemoglobin production in thalassemia respectively.^30^ Abnormal formation of haemoglobin eventually leads to disruption of oxygen transport, destruction of red blood cells and anaemia. Previous studies have reported that anaemia is associated with increased mortality and morbidity in heart failure,^31^ angina pectoris,^32^ acute coronary syndrome,^33^ cancer^34^ and human immunodeficiency virus infection.^35^ In patients with AF, anaemia was an independent predictor of one‐year survival and rehospitalization.^36^ Chronic anaemia can cause many physiological changes in the circulatory system. Low blood viscosity and hypoxic vasodilation contribute to low peripheral resistance. Hypoxemia directly stimulates chemoreceptors in the carotid body, precipitating increased ventilation and sympathetic discharges.^37^, ^38^ Along with sympathetic excitement, chronic anaemia leads to an increase in cardiac output, leading to left ventricular (LV) remodelling.^39^ This might explain why Hb levels increase the risk of AF. Katayama et al. showed that haemodynamic changes due to low or high Hb levels can affect LA remodelling and the development of AF.^40^ In addition, oxidative stress secondary to hypoxia promotes activation of fibroblasts to myofibroblasts, leading to perivascular and interstitial fibrosis, which leads to slow and heterogeneous conduction.^41^ One study showed that nitrate‐functionalized patches confer cardioprotection and improve heart repair after myocardial infarction via local nitric oxide delivery.^42^

SNCA in blood has been tested as a biomarker for Parkinson's disease (PD).^43^, ^44^ It was found that mice expressing the mutant forms of SNCA caused familial PD to exhibit aberrant autonomic control of the heart, characterized by elevated resting heart rate and impaired cardiovascular stress response.^45^ Beatriz Tijero.el found that sympathetic denervation caused by *SNCA* mutations appears to be organ‐specific, and selectively affects the heart.^46^ In our study, we found that *SNCA* was highly expressed in patients with AF and stroke. We speculate that SNCA can act on adrenergic receptors by regulating sympathetic nerve excitation, releasing norepinephrine and causing corresponding changes in various ion channels. This shortens the effective atrial refractory period, increases the dispersion of the effective atrial refractory period and induces AF, which in turn induces sympathetic nerve remodelling, increasing the density and heterogeneity of sympathetic nerves in the atrium. This ultimately leads to a decrease in the expression of the ion channel proteins and a decrease in ion current density, maintaining the action potential cycle and shortening the effective refractory period. Because of this, the AF arrhythmia persists, which in turn leads to the formation and transfer of intra‐atrial thrombus, and ultimately to stroke.

Glycophorin B, a glycoprotein occurring in high levels on the surface of red blood cells, is a receptor for *Plasmodium*, the pathogen causing malaria, and is therefore a key determinant of *Plasmodium* invasion.^47^ The glycophorin gene locus consists of three ~120 kb tandem repeats sharing ~97% identity, each repeat carrying a closely related glycophorin gene, starting from the centromeric end: glycophorin E (*GYPE*), glycophorin B (*GYPB*) and glycophorin A (*GYPA*).^48^ Mutations in the human glycoprotein gene have been reported to reduce the risk of severe malaria and prevent malarial anaemia.^49^ Native myeloperoxidase (MPO) binds specifically to the major integral proteins of RBCs (GYPB), inducing multiple changes in the biophysical properties of the cells. These include the MPO‐induced ‘freezing’ of membrane lipids, transmembrane potential, dynamic changes in the morphology and size of RBCs, increased sensitivity to acidic and osmotic haemolysis, and decrease in cellular deformability. The main function of RBCs is oxygenation of cells and tissues, which largely depends not only on the ability of haemoglobin to bind to and release oxygen, but also on the rheological properties of RBCs (RBC deformability). Decreases in RBC deformability have been well documented in a variety of diseases, including atherosclerosis, ischaemic heart disease, sepsis and diabetes.^50^, ^51^, ^52^ We found that *GYPB* was highly expressed in AF patients with cerebral infarction, compared to AF patients alone. Therefore, we speculate that GYPB promotes atrial thrombosis and metastasis by regulating biophysical properties such as cell membrane fluidity, transmembrane potential, intracellular calcium ions, cell size and morphology, haemolysis sensitivity, and cell deformability, ultimately leading to stroke.

Our study has several limitations. First, this study was conducted to investigate the molecular mechanisms underlying AF. However, comprehensive verification of the conclusions of this study using animal experiments is needed. Second, the sample size was small, so a multi‐centre, randomized, controlled trial study should be carried out in future to validate our results.

In summary, the hub genes *HBG1*, *SNCA* and *GYPB* might be significantly related to AF. These genes are involved in the incidence of AF complicated by stroke by affecting multiple signalling pathways, which may serve as targets for early diagnosis or treatment. Our study provides new evidence and ideas for further exploration of the underlying mechanism and treatment of AF complicated by stroke.

## CONFLICT OF INTEREST

The authors declare that they have no conflict of interest.

## AUTHOR CONTRIBUTION

**Xiang Wang:** Data curation (supporting); Formal analysis (lead); Methodology (lead); Writing – original draft (lead); Writing – review & editing (equal). **Xuyang Meng:** Data curation (supporting); Resources (equal); Supervision (equal). **Ling‐bing Meng:** Data curation (equal); Methodology (equal); Software (equal); Supervision (equal). **Ying Guo:** Investigation (equal); Methodology (equal). **Yi Li:** Resources (equal); Validation (equal). **Chenguang Yang:** Investigation (equal); Resources (equal). **Zuowei Pei:** Investigation (equal); Writing – review & editing (equal). **Jiahan Li:** Software (equal); Supervision (equal). **Fang Wang:** Conceptualization (lead); Data curation (equal); Resources (lead).

## 5 CONSENT FOR PUBLICATION

All the authors have read and approved the current version of the manuscript.

## Supporting information

Supplementary Material

## Data Availability

The data used to support the findings of this study are available from the corresponding author upon request.
