# Supplementary material for: Joint efficacy of the three biomarkers SNCA, GYPB and HBG1 for atrial fibrillation and stroke: Analysis via the support vector machine neural network
Source: J Cell Mol Med. 2022 Feb 9;26(7):2010–22. doi: 10.1111/jcmm.17224 (PMC8980947; doi:10.1111/jcmm.17224)
Supplement: Supplementary file 1 — Supplementary Material [file JCMM-26-2010-s001.docx]

**Results**

**Functional and pathway enrichment analysis of DEGs**

The enrichment results of the GO and KEGG analyses of DEGs performed by BINGO were mainly enriched in “hemoglobin metabolic process”, “oxygen transport”, “cellular homeostasis”, “oxygen homeostasis”, “negative regulation of oxidoreductase activity”, “oxygen transporter activity”, “oxygen binding” (Figure S1A-B).

The enrichment results of the GO and KEGG analyses of DEGs performed by Metascape were mainly enriched in “hydrogen peroxide catabolic process”, “cell-cell junction organization”, “positive regulation of protein-containing complex assembly” in the bar diagram (FigureS2 A), and interactive network diagram (FigureS2 B), and the P-value map (FigureS2 C). Summary of enrichment analysis in DisGeNET presented that the DEGs were mainly enriched in the “Hemoglobinopathies”, “Abnormal hemoglobin finding” (FigureS2 D). Furthermore, the main enriched cells were “CD71^+^”, “Macrophage cell”, and “K562” (FigureS 2E).

Top 6 clusters with their representative enriched terms (one per cluster) via Metascape were showed in the Table S2. Summary of enrichment analysis in DisGeNET was presented in the Table S3. Summary of enrichment analysis in PaGenBase was manifested in the TableS4.

**GSEA analysis for the GO enrichment**

The enrichment results of the GO analysis of DEGs were obtained using GSEA; they were mainly enriched in left ventricular hypertrophy, immunoglobulin production, immunoglobulin complex circulating (FigureS3 A-C). The comparison between AF and control was fine by the enrichment score, significance, ranked gene list correlation profile (FigureS3 D-F).

**The DEGs between AF with stroke (AFST) and the control group**

The volcano map manifested numerous DEGs between AF with stroke and the control group (FigureS4A). The venn diagram identified the common significant genes between “AFST. vs. Con” and “AF.vs.Con”, including SNCA, GYPB (FigureS4 B).

**Validation of the dataset of GSE75092**

Through the PCA analysis, the intra-group data repeatability is fine in the GSE75092. The distances between per samples in the control group were close, and distances between per samples in the AF group were also close in the dimension of PC1 (Figure S4C). Furthermore, based on the Pearson’s correlation test, we found that in the GSE75092, there exists strong correlation among the samples in the AF, and there exists strong correlations among the samples in the control (Figure S4D).

**Differently expressed LincRNAs between AF and control groups**

Between the AF and control groups, the heatmap manifested the expression level of differently expressed LincRNAs, and there existed significant differences (Figure S4E).

Through the volcano map, there were numerous differently expressed LincRNAs between the AF and control group, and the green plots presented the down-regulated differently expressed LincRNAs and the red plots presents the up-regulated differently expressed LincRNAs (Figure S4F).


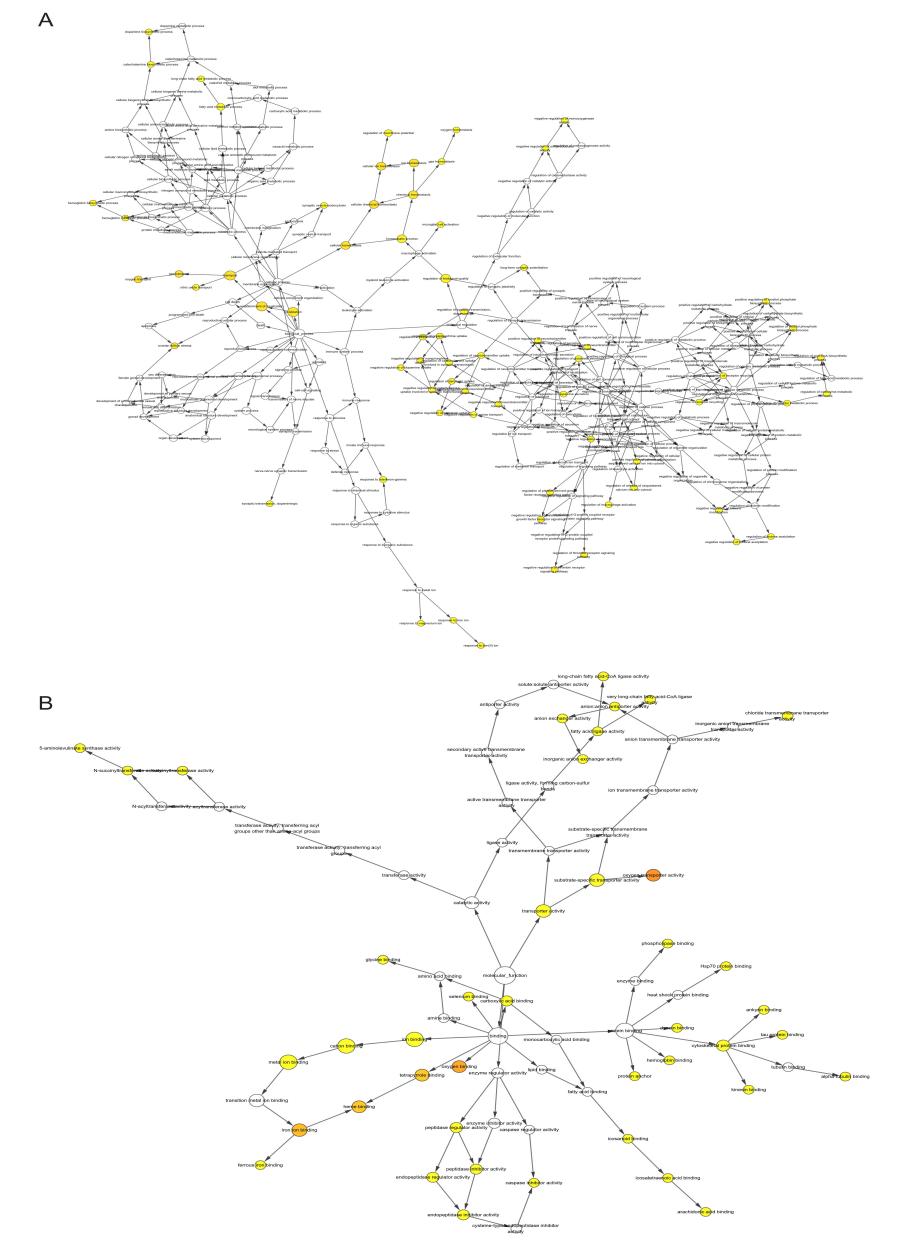


Figure S1. The enrichment results of the GO and KEGG analyses of DEGs performed by BINGO


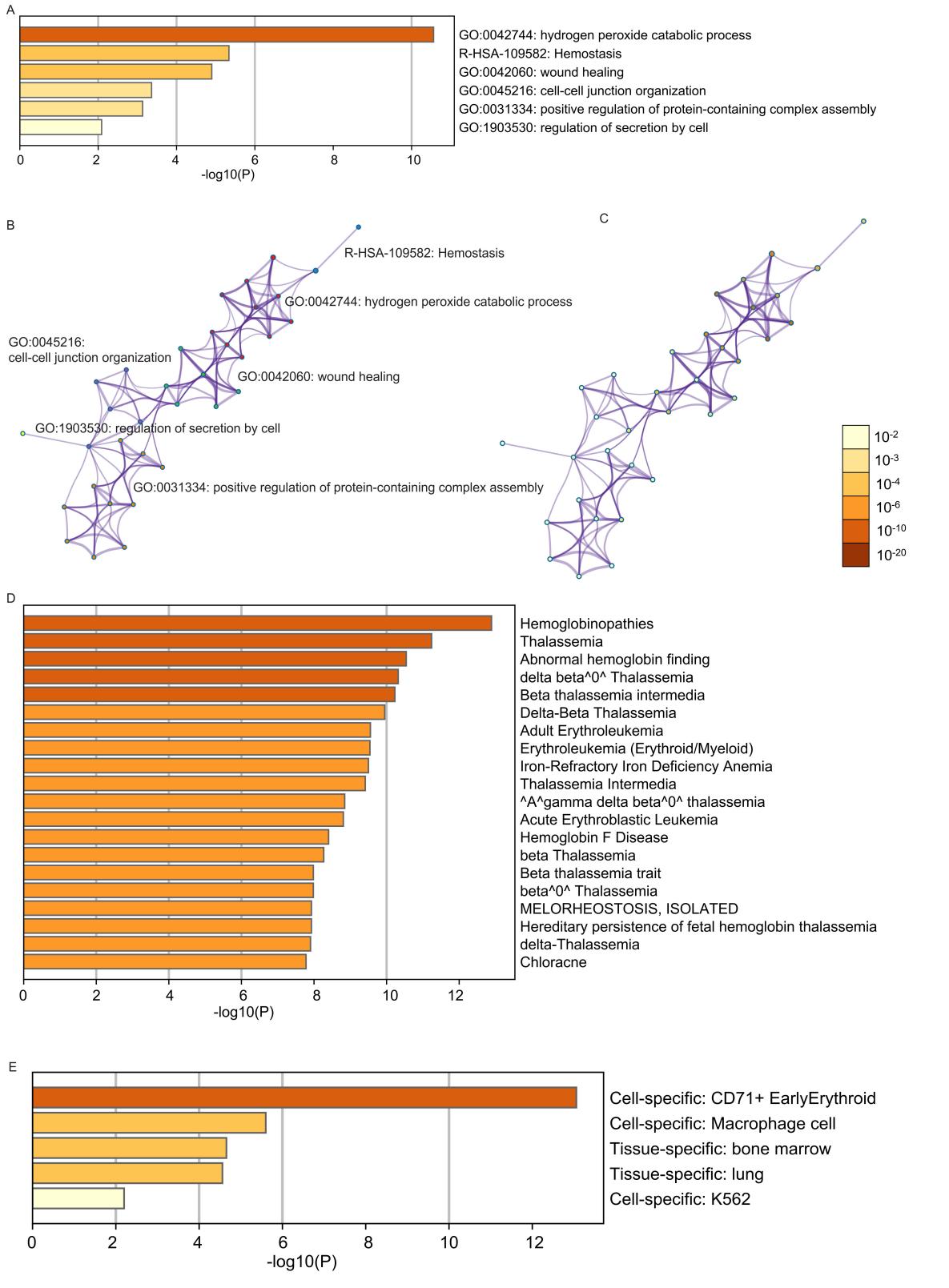


Figure S2. The enrichment results of the GO and KEGG analyses of DEGs performed by Metascape


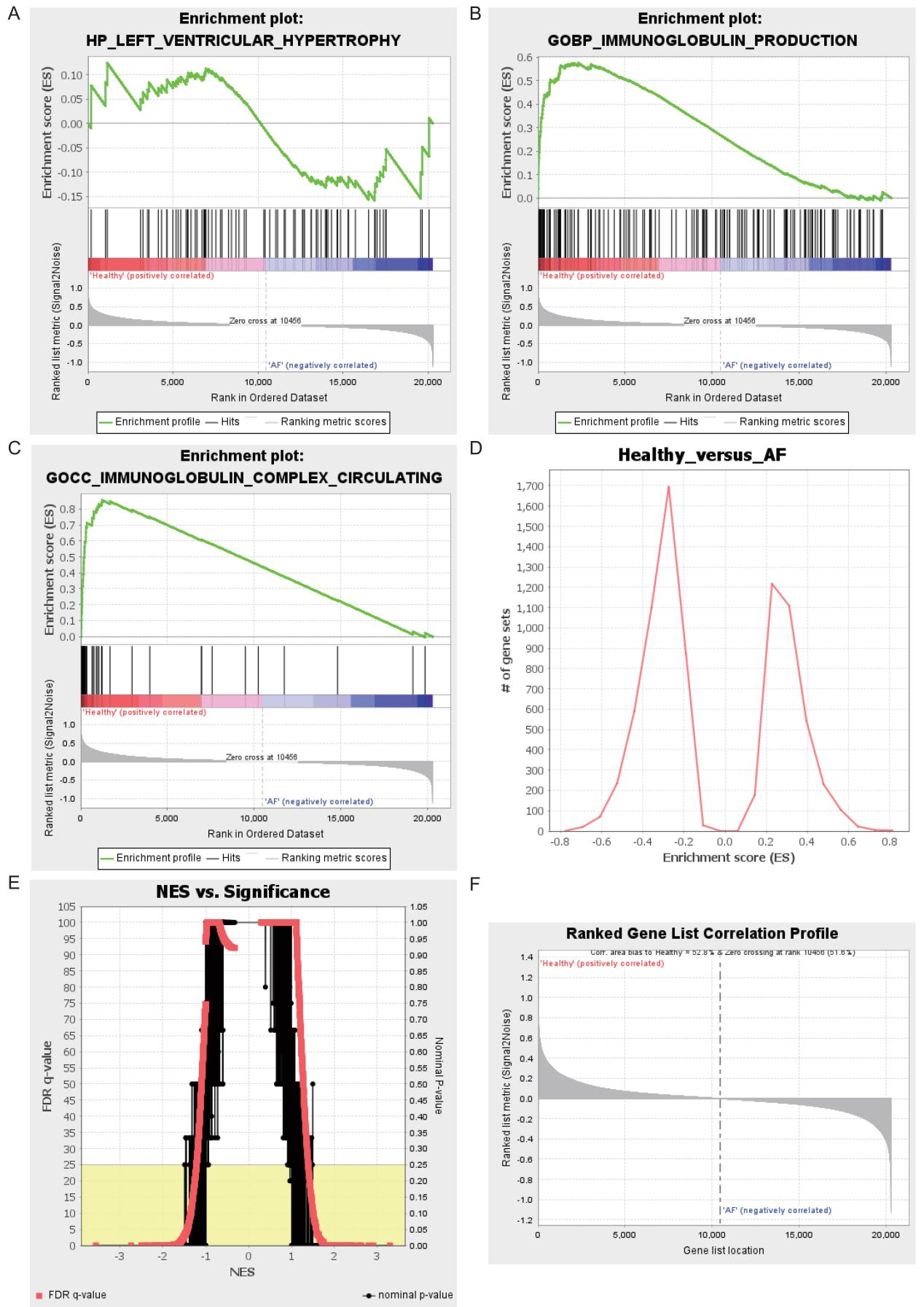


Figure S3. GSEA analysis for the GO enrichment


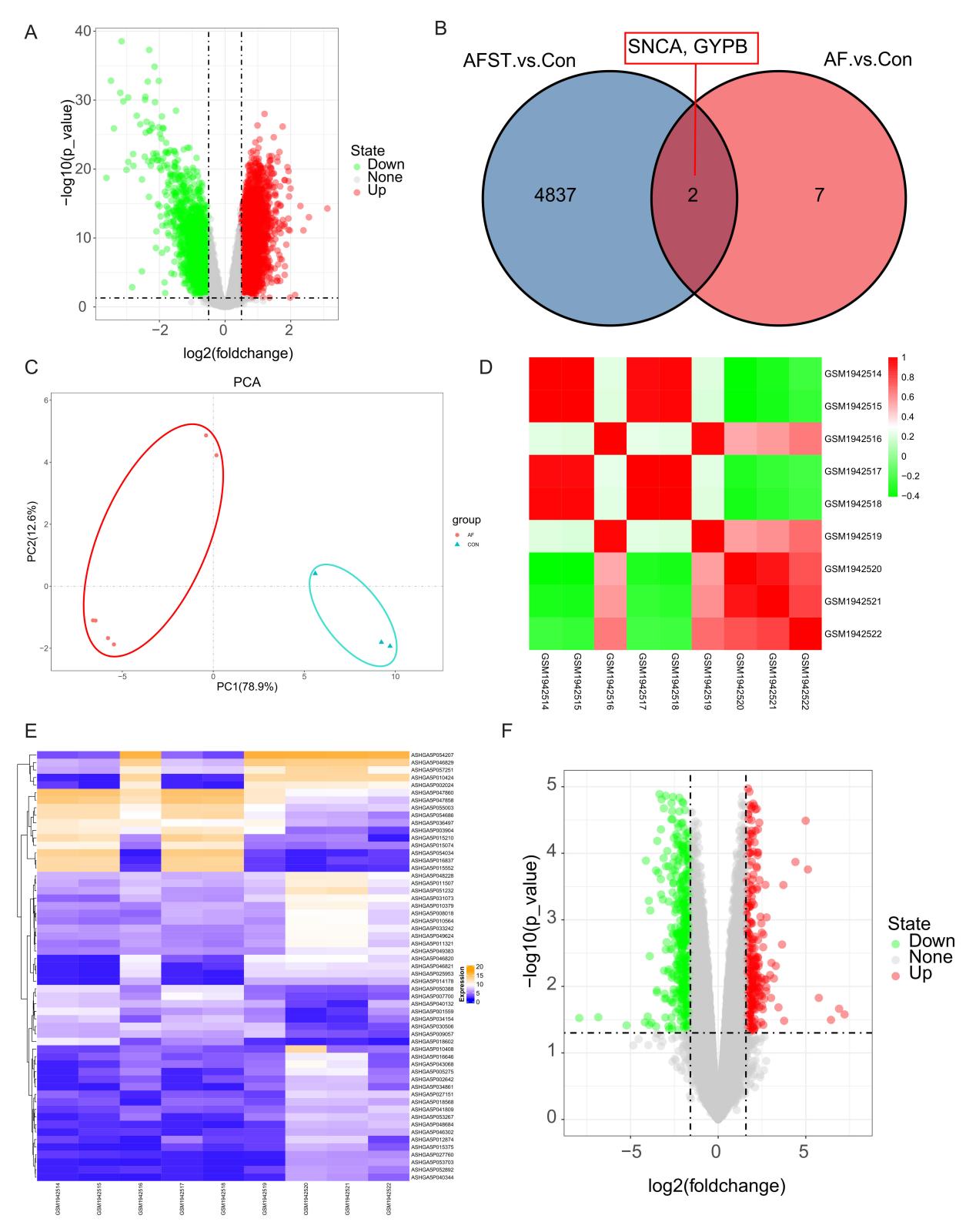


Figure S4. The DEGs between AF with stroke (AFST) and the control group, and validation of the dataset of GSE75092

| Table S1. Primers of the genes | |
| --- | --- |
| Gene name | Primer(5'-3') |
| H-HBG1-S | ATGGGTCATTTCACAGAGGAGG |
| H-HBG1-A | ATGGGTAGACAACCAGGAGCC |
|  |  |
| H-HBD-S | TGCCTTTAGTGATGGCCTGG |
| H-HBD-A | AACAGTCCAGGATCTCAATGGT |
|  |  |
| H-ALAS2-S | GTCTCAGTCAGCCTAAGGAAGCC |
| H-ALAS2-A | GAGAAATGTTGGGCAAAGGGA |
|  |  |
| H-SNCA-S | CAGCAGTAGCCCAGAAGACAGT |
| H-SNCA-A | GGCATTTCATAAGCCTCATTGTC |
|  |  |
| H-CYPB-S | CTCCGAACGCAACATGAAGG |
| H-CYPB-A | CCAAAGATCACCCGGCCTAC |
|  |  |
| HBA2-S | CCTACTTCCCGCACTTCGA |
| HBA2-A | GGCTCCAGCTTAACGGTATTTG |
|  |  |
| SELENBP1-S | GGGTGGAGGGACTTCAGA |
| SELENBP1-A | CGCCTAACCTGGCAATAC |
|  |  |
| SLC4A1-S | GGGACGGTCAGGTCAATA |
| SLC4A1-A | ATGGGAAAGAACGGAGGA |
|  |  |
| HBB-S | CCCAGAGGTTCTTTGAGT |
| HBB-A | AGCGTCCCATAGACTCAC |

| Table S2. Top 6 clusters with their representative enriched terms (one per cluster). | | | | | | |
| --- | --- | --- | --- | --- | --- | --- |
| **GO** | **Category** | **Description** | **Count** | **%** | **Log10(P)** | **Log10(q)** |
| GO:0042744 | GO Biological Processes | hydrogen peroxide catabolic process | 5 | 23.81 | -10.56 | -6.20 |
| R-HSA-109582 | Reactome Gene Sets | Hemostasis | 6 | 28.57 | -5.34 | -2.09 |
| GO:0042060 | GO Biological Processes | wound healing | 5 | 23.81 | -4.90 | -1.74 |
| GO:0045216 | GO Biological Processes | cell-cell junction organization | 3 | 14.29 | -3.36 | -0.32 |
| GO:0031334 | GO Biological Processes | positive regulation of protein-containing complex assembly | 3 | 14.29 | -3.13 | -0.17 |

| Table S3: Summary of enrichment analysis in DisGeNET. | | | | | |
| --- | --- | --- | --- | --- | --- |
| **GO** | **Description** | **Count** | **%** | **Log10(P)** | **Log10(q)** |
| [C0019045](http://www.disgenet.org/browser/0/0/3/0/diseaseid__C0019045-source__ALL/_b./) | Hemoglobinopathies | 7 | 33 | -13.00 | -8.70 |
| [C0039730](http://www.disgenet.org/browser/0/0/3/0/diseaseid__C0039730-source__ALL/_b./) | Thalassemia | 7 | 33 | -11.00 | -7.20 |
| [C0349705](http://www.disgenet.org/browser/0/0/3/0/diseaseid__C0349705-source__ALL/_b./) | Abnormal hemoglobin finding | 4 | 19 | -11.00 | -6.60 |
| [C0271986](http://www.disgenet.org/browser/0/0/3/0/diseaseid__C0271986-source__ALL/_b./) | delta beta Thalassemia | 4 | 19 | -10.00 | -6.50 |
| [C0472767](http://www.disgenet.org/browser/0/0/3/0/diseaseid__C0472767-source__ALL/_b./) | Beta thalassemia intermedia | 5 | 24 | -10.00 | -6.50 |
| [C0271985](http://www.disgenet.org/browser/0/0/3/0/diseaseid__C0271985-source__ALL/_b./) | Delta-Beta Thalassemia | 4 | 19 | -9.90 | -6.30 |
| [C2347748](http://www.disgenet.org/browser/0/0/3/0/diseaseid__C2347748-source__ALL/_b./) | Adult Erythroleukemia | 7 | 33 | -9.60 | -6.00 |
| [C4520840](http://www.disgenet.org/browser/0/0/3/0/diseaseid__C4520840-source__ALL/_b./) | Erythroleukemia (Erythroid/Myeloid) | 7 | 33 | -9.50 | -6.00 |
| [C0085576](http://www.disgenet.org/browser/0/0/3/0/diseaseid__C0085576-source__ALL/_b./) | Iron-Refractory Iron Deficiency Anemia | 5 | 24 | -9.50 | -6.00 |
| [C0271979](http://www.disgenet.org/browser/0/0/3/0/diseaseid__C0271979-source__ALL/_b./) | Thalassemia Intermedia | 5 | 24 | -9.40 | -5.90 |
| [C0271987](http://www.disgenet.org/browser/0/0/3/0/diseaseid__C0271987-source__ALL/_b./) | gamma delta beta thalassemia | 3 | 14 | -8.80 | -5.50 |
| [C0023440](http://www.disgenet.org/browser/0/0/3/0/diseaseid__C0023440-source__ALL/_b./) | Acute Erythroblastic Leukemia | 7 | 33 | -8.80 | -5.40 |
| [C0019025](http://www.disgenet.org/browser/0/0/3/0/diseaseid__C0019025-source__ALL/_b./) | Hemoglobin F Disease | 4 | 19 | -8.40 | -5.10 |
| [C0005283](http://www.disgenet.org/browser/0/0/3/0/diseaseid__C0005283-source__ALL/_b./) | beta Thalassemia | 6 | 29 | -8.30 | -5.00 |
| [C0271980](http://www.disgenet.org/browser/0/0/3/0/diseaseid__C0271980-source__ALL/_b./) | beta Thalassemia | 4 | 19 | -8.00 | -4.70 |
| [C0878521](http://www.disgenet.org/browser/0/0/3/0/diseaseid__C0878521-source__ALL/_b./) | Beta thalassemia trait | 4 | 19 | -8.00 | -4.70 |
| [C0271994](http://www.disgenet.org/browser/0/0/3/0/diseaseid__C0271994-source__ALL/_b./) | Hereditary persistence of fetal hemoglobin thalassemia | 4 | 19 | -7.90 | -4.70 |
| [C3149631](http://www.disgenet.org/browser/0/0/3/0/diseaseid__C3149631-source__ALL/_b./) | MELORHEOSTOSIS, ISOLATED | 4 | 19 | -7.90 | -4.70 |
| [C0271990](http://www.disgenet.org/browser/0/0/3/0/diseaseid__C0271990-source__ALL/_b./) | delta-Thalassemia | 3 | 14 | -7.90 | -4.70 |
| [C0263454](http://www.disgenet.org/browser/0/0/3/0/diseaseid__C0263454-source__ALL/_b./) | Chloracne | 4 | 19 | -7.80 | -4.60 |

| Table S4: Summary of enrichment analysis in PaGenBase. | | | | | |
| --- | --- | --- | --- | --- | --- |
| **GO** | **Description** | **Count** | **%** | **Log10(P)** | **Log10(q)** |
| PGB:00026 | Cell-specific: CD71+ EarlyErythroid | 8 | 38 | -13.00 | -8.70 |
| PGB:00058 | Cell-specific: Macrophage cell | 3 | 14 | -5.60 | -2.70 |
| PGB:00048 | Tissue-specific: bone marrow | 4 | 19 | -4.70 | -1.80 |
| PGB:00018 | Tissue-specific: lung | 5 | 24 | -4.60 | -1.70 |
| PGB:00027 | Cell-specific: K562 | 3 | 14 | -2.20 | 0.00 |
